# Virtual Northern Analysis of the Human Genome

**DOI:** 10.1371/journal.pone.0000460

**Published:** 2007-05-23

**Authors:** Evan H. Hurowitz, Iddo Drori, Victoria C. Stodden, David L. Donoho, Patrick O. Brown

**Affiliations:** 1 Department of Biochemistry, Stanford University School of Medicine, Stanford, California, United States of America; 2 Department of Statistics, Stanford University, Stanford, California, United States of America; 3 Howard Hughes Medical Institute, Stanford University School of Medicine, Stanford, California, United States of America; Centre de Regulació Genòmica, Spain

## Abstract

**Background:**

We applied the Virtual Northern technique to human brain mRNA to systematically measure human mRNA transcript lengths on a genome-wide scale.

**Methodology/Principal Findings:**

We used separation by gel electrophoresis followed by hybridization to cDNA microarrays to measure 8,774 mRNA transcript lengths representing at least 6,238 genes at high (>90%) confidence. By comparing these transcript lengths to the Refseq and H-Invitational full-length cDNA databases, we found that nearly half of our measurements appeared to represent novel transcript variants. Comparison of length measurements determined by hybridization to different cDNAs derived from the same gene identified clones that potentially correspond to alternative transcript variants. We observed a close linear relationship between ORF and mRNA lengths in human mRNAs, identical in form to the relationship we had previously identified in yeast. Some functional classes of protein are encoded by mRNAs whose untranslated regions (UTRs) tend to be longer or shorter than average; these functional classes were similar in both human and yeast.

**Conclusions/Significance:**

Human transcript diversity is extensive and largely unannotated. Our length dataset can be used as a new criterion for judging the completeness of cDNAs and annotating mRNA sequences. Similar relationships between the lengths of the UTRs in human and yeast mRNAs and the functions of the proteins they encode suggest that UTR sequences serve an important regulatory role among eukaryotes.

## Introduction

Now that the human genome sequence is nearly complete [1]–[3], the next step is to characterize the organization, function, and diversity of the human genome. Reliable computational detection and analysis of genes in mammalian genomes remains a challenge due to the low percentage of coding sequence, the existence of many short exons and long introns, and the high diversity of alternate transcript forms [1]. Therefore, most efforts to annotate the human genome have relied heavily on the analysis of expressed sequences generated from human RNA. Recently however, the focus has shifted from the generation of ESTs, which are generally short clones representing a fraction of their parent transcript, to the generation of full-length cDNAs. Due to a number of large-scale full-length cDNA sequencing projects, over 20,000 human genes have been validated by at least one putative full-length cDNA [4].

Although full-length cDNA sequencing projects provide the basis for virtually all human gene identification and analysis, they suffer from several limitations. First, they are expensive and labor-intensive. Second, there are no fool-proof methods for cloning only full-length cDNAs, or identifying cDNA clones that are not full-length. There are a number of well-accepted cloning methods that enrich for full-length cDNAs [4], but these methods may yield the true 5′-end only 80% of the time [5]. Methods for identifying cDNA clones that are not full-length typically involve either comparison of the clones to other clones, computational analysis of the cDNA's sequence to identify a translational initiation site, or computational analysis of the genome sequence upstream from the cDNA to identify a putative promoter [6]. Although all of these approaches are valid and important analyses, none of them actually ensure that the clone is full-length, especially in cases where the full transcript may be difficult to clone, for example, due to secondary structure in the transcript. Third, long transcripts are under-represented in cDNA clone libraries. Finally and most importantly, full-length cDNA projects suffer from a strong sampling bias due to very large differences in expression levels between different transcripts. For that reason, only the most abundantly expressed transcripts are well-sampled in cDNA libraries. Most genes are represented in these libraries by fewer than two full-length transcripts [4], allowing many inabundant transcripts and transcript variants to go undetected [7]. Furthermore, the small numbers of cDNA clones representing most genes makes estimates of the relative abundance of transcripts from tissue to tissue, and variant to variant, unreliable. Due to these limitations, it is unlikely that the goal of completely characterizing the human transcriptome, including all transcript variants across all tissues, disease states, and developmental stages, will be accomplished by full-length cDNA sequencing alone.

Characterization of RNA transcripts by length does not have the resolution to identify precise sites of transcript initiation or termination, precise splice sites, or even exon-intron structure, but it does provide an unbiased measurement of transcript length, a quantity that is relatively difficult to obtain through full-length cDNA sequencing alone. This independent characterization of mRNA length is useful in determining if clones are in fact full-length, and provides an additional parameter for computationally identifying genes from the genome sequence. By comparing our length measurements to the Unigene, Refseq [8], and H-Inv [4] databases, our measurements allowed us to evaluate current progress in cataloging the transcriptome.

## Results

### Evaluation of the human Virtual Northern

**Figure 1:**
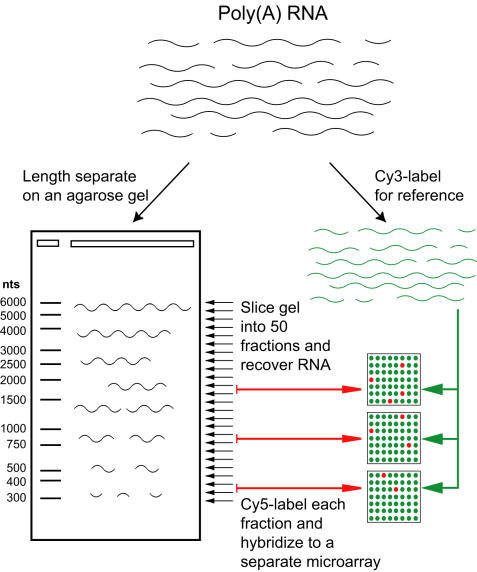
Virtual Northern scheme.

**Figure 2:**
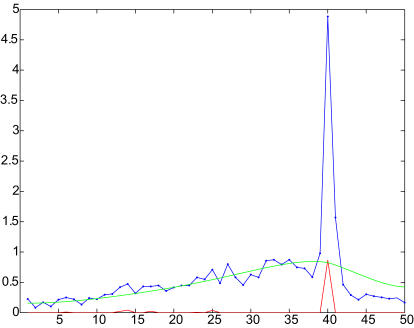
Example length profile with deconvolution results. An example length profile is shown in blue. The normalized ratio from each length fraction is plotted against the length fractions in order, where the first length fraction is the one with the highest gel mobility. The rolling baseline is shown in green, and the deconvolution result is shown in red.

We applied the Virtual Northern technique [9] to the human genome in order to further characterize the human transcriptome. Virtual Northern analysis uses gel electrophoretic separation of mRNAs by length, and DNA microarray analysis to read out the results for a large set of genes in parallel. Briefly, we separated human brain mRNA by length on an agarose gel, sliced the gel into 50 narrow sections each containing RNA from a small range of lengths, and hybridized the RNA from each slice to a separate cDNA microarray (Figure 1). The data for each cDNA from all 50 microarrays were combined into a profile that peaks in the slice, or slices, that contain mRNAs complementary to a given cDNA sequence represented on the microarray (Figure 2). We searched each length profile for peaks by *l*_1_ norm baseline deconvolution, and estimated a confidence value for each peak with a bootstrapping approach. The experiment was performed in triplicate and the results from all three replicates were combined to create a dataset of peaks along with their associated confidence values for each cDNA. Concordance between the three replicates was strongly correlated with microarray signal strength, ranging from virtually 100% for the most strongly expressed genes to almost 0% for the most weakly expressed genes. Replication of the experiment greatly reduced the noise, and increased confidence of many weak but legitimate peaks. Using a set of well-studied (“gold standard”) genes with mRNAs of known length, we converted the locations of the peaks within each profile into transcript lengths in nucleotides. The conversion explicitly accounted for the length of each transcript's poly(A) tail by adding a fixed length to each known length. A fixed length of 225 nucleotides was calculated to provide the best fit between the peak locations and the known transcript lengths. We also explicitly calculated our accuracy, allowing us to calibrate our bootstrap values to the probability that an observed peak represents a real transcript (the true positive rate).

The resulting dataset contained 21,933 combined length profiles with peaks ranging in confidence from 6% to 100%. At a high stringency 90% confidence cutoff, we identified 8,774 clones with at least one length, and a mean confidence for all peaks of 97% (Table 1). Of those 8,774 clones, 118 (1.3%) had a two peaks. The mean and median of all lengths at 90% were 2,165 and 1,996 nucleotides respectively. These values are similar to a poly(A) tail-corrected mean of 2,635 nucleotides and median of 1,965 nucleotides previously estimated for the human genome [1].

**Table 1:** Results of data filtering and peak finding

Spot type	Number of spots on each array	Number of profiles with at least one peak of 90% confidence
Human	41418 (39355)	8774 (7989)
*M. jannaschii*	320 (40)	189 (26)
Nonhuman	175 (89)	0
Total	41913 (39484)	8963 (8015)

Since these microarrays contained duplicated spots, the parentheses represent the number of unique spots or profiles in the dataset.

Using information downloaded from the National Center for Biotechnology Information (NCBI) website (http://www.ncbi.nlm.nih.gov/Ftp/) on August 8, 2005, each cDNA clone spotted on the microarrays could be associated with the human gene or genomic locus from which it derives [8]. Out of 41,418 cDNA clones on the microarrays, 31,708 (76.6%) of them could be assigned to at least one Entrez gene (Table 2). The remaining 9,710 clones with no assignment to an Entrez gene represent putative expressed sequences whose cognate genes remain uncharacterized. Since multiple clones can map to the same locus, the 31,708 clones represent 15,552 distinct genes. We identified a transcript at ≥90% confidence for 6,238 (40%) of the 15,552 currently annotated human genes represented on the microarrays. As shown in Table 2, the percentage of clones for which we identified a peak was much lower among the clones with no assignment to an Entrez gene than those with a gene assignment. This probably reflects the enrichment in this set of clones for cloning artifacts, or rarely expressed genes that would be difficult to detect by either hybridization or cDNA cloning based methods.

**Table 2:** Association between cDNA clones and human genes

Number of human clones with a peak of at least % confidence	90%	Whole array
Assigned to at least one human locus	7905 (7146)	31708 (29972)
Assigned to only one human locus	6819 (6485)	28244 (27055)
Not assigned	869 (843)	9710 (9383)
Total	8774 (7989)	41418 (39355)

Since these microarrays contained duplicated spots, the parentheses represent clones after averaging.

**Figure 3:**
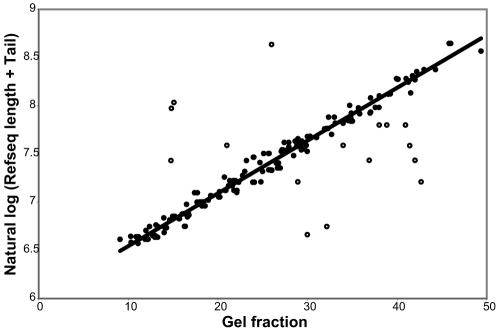
Calibrating the relationship between gel mobility and transcript length. The precise gel mobilities of all peaks from gold standard genes are plotted against the natural log of the sum of their matching Refseq length and an estimated poly(A) tail length of 225 nucleotides. The least squares fit to a line is shown by a black line with the parameters y = 0.054731 x+5.997276 (R^2^ = 0.99). Closed circles represent points used to determine the calibration line. Points shown by open circles were excluded from the least squares calculation.

The precision with which we measured human transcript lengths was estimated based on the deviations between transcript lengths inferred from the Virtual Northern method and the lengths of the mRNAs in our “gold standard” set (see Materials and Methods), and on the variation among multiple measurements for the same gene. As we previously found in our analysis of yeast transcripts, one major limitation of precision was the precision with which the gel was sliced [9]. In this case, due to the approximately exponential relationship between gel mobility and RNA length, the range of lengths represented by each 2 mm slice increased from 24 to 331 nucleotides with decreasing distance from the origin over the range analyzed. However, as a fraction of RNA length the variation represented in each slice remained a constant 5.6%. Nevertheless, the average absolute deviation between 184 Virtual Northern measurements and the length of their matching gold standard mRNA was 12.2%. This estimation of precision is twice the width of each slice, but it is likely an overestimate of the true deviation. As shown in Figure 3, sixteen outliers were excluded from the calculation of the best fit line. With those outliers also excluded from the precision calculation, the average absolute deviation becomes 5.5%. We therefore consider 5.5% (approximately the gel slice width) to be a more accurate estimate of our measurement precision since the larger estimate is probably inflated due to the inclusion of false positive measurements. Additionally, the deviations between multiple measurements for the same gene were calculated. That average standard deviation was calculated to be only 2.7% over 1,846 combined profiles representing 492 unique sequences represented on the microarrays. Since the variations among replicate measurements are substantially less than the imprecision in the transformation of gel mobility to mRNA length, we conclude that the measurement precision is 5.5%, approximately the gel slice width.

### Unigene Cluster Analysis

Many of the distinct clones in our dataset for which we measured a transcript length are associated with the same Unigene cluster. We examined all such clusters to determine if the length measurements from all clones associated with the same cluster were the same. Distinct clones belonging to the same Unigene cluster can sometimes detect alternative transcripts with distinct expression patterns (Jeremy Gollub, personal communication). This discordant behavior can be caused by a number of technical factors including chimeric or misidentified clones, Unigene cluster or human genome assembly errors, or cross-hybridization artifacts. A more interesting cause of discordant expression patterns is the inclusion of clones in the same Unigene cluster that inadvertently represent different transcript variants of the same gene.

We began by analyzing the transcipt length measurements derived from microarray hybridization data for the 1,236 cDNA clones that map to more than one Unigene cluster. Clones can map to as many as three clusters due to any of the factors discussed above. We compared the length measurements for each of the 1,236 clones to the length measurements of all of the other clones in our dataset that map to any of the same clusters. For many of the clusters in this set, there were no other clones in our dataset that mapped to the same cluster, so in many cases we could only compare a clone to a subset of the clusters to which it belongs. We performed this analysis on lengths of 34%, 50%, and 90% confidence, but obtained similar results in each case. The following represents the analysis of the 90% confidence lengths. For 217 clones, we could compare the clone to only one cluster. In 108 (50%) of those cases, the clone matched (*i.e.* shared a length in common with) that cluster. For 115 clones, we compared the clone to two clusters. In 61 (53%) of those cases the clone matched only one cluster, in another 29 (25%) of those cases the clone matched both clusters, and in 25 cases (22%) it matched neither. For the two clones that we could compare to three clusters, one matched one of the three clusters and the other matched two. Although we were not able to compare each clone to each of the clusters to which it belongs, there is clearly a high prevalence of both clones that match only a single cluster and clones that match multiple clusters. Therefore, the set of clones that map to multiple Unigene clusters are not simply incorrectly assigned to multiple clusters. Many appear to hybridize to mRNAs transcribed from multiple genes.

We then analyzed 4,883 Unigene clusters with an average of 2.6 clones per cluster (Table 3). Excluding clones that mapped to multiple Unigene clusters, we grouped the clones from each cluster into subclusters based on whether or not the microarray hybridization results derived from those clones yielded the same transcript length measurements. Clones yielding concordant transcript lengths were placed in the same subcluster while clones yielding discordant transcript lengths were placed in separate subclusters. Using lengths of at least 90% confidence, 28% of the Unigene clusters were broken up into 2 to 3 subclusters.

**Table 3:** Unigene cluster analysis

Number of clones/cluster	Number of clusters
2	3027
3	1168
4	437
5	170
6	41
7	19
8	13
9	3
10	2
11	2
12	1
Total	4883

We also identified groups of clones from the same Unigene cluster containing overlapping sequence. Using information available from the UCSC genome browser [10], we determined the genome coordinates of the cDNA clones in our dataset. Excluding clones that mapped to multiple locations in the genome, we subclustered clones belonging to the same Unigene cluster on the basis of whether or not their genome coordinates were overlapping. With the 90% confidence length set, 67% of the Unigene clusters were broken up into 2 to 5 subclusters based on their genome coordinates.

**Figure 4:**
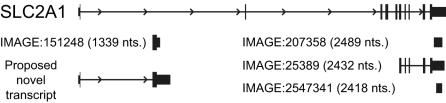
Solute carrier family 2 (facilitated glucose transporter), member 1 (*SLC2A1*) gene. The *SLC2A1* gene is pictured schematically. The transcribed portion of the gene is shown with the filled boxes representing exons. The ORF is represented by the taller boxes. The genomic positions of four cDNA clones that map only to the *SLC2A1* gene, and a proposed novel *SLC2A1* transcript, are shown relative to the *SLC2A1* gene. The transcript length measured for each clone is shown in parentheses.

We then compared the length-based subclusters to the coordinate-based subclusters in order to explore the possibility that the length-based subclusters represent alternate transcript variants that are being detected by clones that map to distinctly different sections of the underlying gene. As a result, we found a high degree of similarity between the patterns of length- and coordinated-based subclustering. With the 90% confidence length set, 64% of the pairs of clones in different length subclusters were also in different coordinate subclusters. This was a significantly higher percentage than would be expected based on chance (chi-squared test, p = 7.2×10^−9^). One interesting result is shown in Figure 4 for four clones representing the glucose transporter *SLC2A1* gene. IMAGE clone 151248 was split from the other two clones in its Unigene cluster on the basis of both length and genome alignment. Although IMAGE clones 207358, 25389, and 2547341 are perfect matches for the 3′-end of the *SLC2A1* gene and identify lengths that agree with the annotated *SLC2A1* transcript, clone 151248 aligns within an intron and identifies a distinctly shorter transcript. Clone 151248 may therefore represent a novel exon of *SLC2A1*, a novel gene residing in *SLC2A1*'s intron, an unspliced *SLC2A1* pre-mRNA species, or even accidentally cloned genomic DNA. Based on additional EST evidence and conservation with multiple other vertebrate genome sequences [10], it appears to represent a novel *SLC2A1* transcript as shown in Figure 4. Likewise, the majority of discrepancies between lengths measured by different clones from the same Unigene cluster that we have identified are due to clones that deviate from the established annotation. Many of them likely represent novel transcripts.

### Comparison of measured transcript length to the Refseq and H-Inv databases

We compared our transcript length dataset to the lengths of the sequences in the human Refseq database. Using information downloaded from the NCBI website (http://www.ncbi.nlm.nih.gov/Ftp/), we were able to associate 17,364 unique clones for which we had a total of 23,324 length measurements with 11,530 Entrez genes, and 15,104 Refseqs. There are more Refseqs than genes, because the Refseq database stores alternate transcripts associated with the same loci, so some genes in the dataset have as many as 21 associated Refseqs.

We compared each of the length measurements in our dataset with each of the Refseqs for the clone's parent gene. We declared a match if our length measurement deviated by no more than three fraction lengths (16.5%) from a poly(A) tail-corrected Refseq length for the same gene. Considering only length measurements of ≥90% confidence, 61% of 7,182 lengths matched a Refseq. In order to determine the significance of the match between Refseq and our measurements, we used a permutation analysis to calculate the match that would occur between our data and Refseq based on chance. The association between genes and Refseq lengths was randomized 10 times, and each time the match between our data and the Refseq lengths of unrelated genes was calculated. The frequency of random matches was consistently about 22% across all permutations at all confidence levels. For the length measurements that did not match their corresponding Refseq lengths, we calculated which was longer. The Refseq was typically longer. At 90% confidence, the Refseq length exceeded our length measurement in 4,378 cases (77%).

It is not entirely surprising that our transcript length dataset matches Refseq only half of the time. If we assume that the human genome encodes 25,000 genes [3], that 74% of all genes are alternately spliced with an average of 2.7 alternate splice forms [11], [1], [4], and that 24% of all genes have alternate polyadenylation sites [1], we would conservatively estimate that the human genome encodes a total of 70,000 different transcripts. As of this writing the Refseq database (Release 12) contains 29,476 mRNA sequences, approximately 42% of the estimated 70,000. Furthermore, the brain is known to be the tissue with the greatest amount of alternative splicing [12], [13]. It is therefore likely that our Virtual Northern experiment has detected many mRNA transcript variants that remain to be annotated in Refseq.

We also compared our transcript length dataset to the lengths of the cDNA clones in the H-Invitational (H-Inv) full-length cDNA database [4]. Release 2 of the H-Inv database contains 56,419 cDNA clones representing a total of 25,585 different loci. Entrez genes were associated with 40,623 of those. We were therefore able to associate 15,403 clones for which we had a total of 20,583 length measurements with 45,005 H-Inv cDNAs. We compared our length measurements to the H-Inv clone lengths just as we compared them to Refseq. The correspondence between our 90% confidence length measurements and the H-Inv cDNAs was 69%. Although we calculated a higher correspondence between our dataset and the H-Inv clones than we did to Refseq, an analogous permutation analysis showed that the probability of a random match was also higher, approximately 35%. Thus, the correspondences between our dataset and Refseq, and our dataset and H-Inv are similar. We also examined the lengths in our dataset that did not match an H-Inv clone to see which was longer. Here, we found an almost 50-50 split across all confidence levels, indicating no systematic difference in lengths between our measurements and the H-Inv clones.

In order to better understand the relationship between our lengths and the Refseq and H-Inv databases, we directly compared the Refseq and H-Inv databases using the same procedure with which we had compared them to our data. The correspondence in length between the two was 58% with a 21% probability of a random match. Thus the correspondence between a well-annotated cDNA clone set and an mRNA transcript database based on cDNA sequence was no greater than the correspondence between either resource and our length dataset. This was somewhat unexpected since it is possible for Refseqs to be based on cDNAs included in the H-Inv clone set. We also found a strong bias for the Refseq to be longer than the H-Inv clone. Nearly 79% of the Refseqs that were not approximately equal in length to their corresponding H-Inv clones were longer. Comparison of Refseqs and available cDNA sequences from the same gene to the genome sequence quickly explained the strong bias toward longer Refseqs. In every case we've examined, the Refseq extends from the 5′-most cDNA to the 3′-most cDNA even when those cDNAs are anomalies that sometimes extend far past the endpoints of virtually all other available cDNA sequences. Thus, there is a tendency for Refseqs to contain additional sequence on their 5′- and 3′-ends beyond what is commonly observed for transcripts from the same gene. It remains to be determined whether this additional sequence represents biologically relevant sequence whose inclusion in the mRNA is rarely needed, or if it represents rare transcriptional accidents.

### Relationship between ORF Length and mRNA length

**Figure 5:**
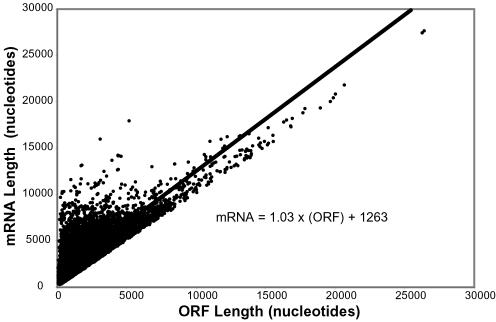
Relationship between ORF length and transcript length. Refseq length in nucleotides is plotted against ORF length in nucleotides. The black line is the linear least squares fit. It has the parameters mRNA = 1.03 (ORF)+1263 (R^2^ = 0.74).

We had previously identified a close linear relationship between open reading frame (ORF) length and mRNA length in the yeast genome [9], so we were interested in determining whether a similar relationship exists for human mRNAs. Since Refseq entries include coding sequence information, we extracted the position of the ORF from every Refseq entry and used that information to calculate the length of the 5′-UTR, ORF, and 3′-UTR for every Refseq. We excluded Refseqs for noncoding RNAs and Refseqs with calculated 5′- or 3′-UTRs less than 20 nucleotides from further analysis (20% of the total). Figure 5 shows a plot of the total length of each human Refseq mRNA versus the length of its ORF. Remarkably, mRNA length shows an excellent fit to a linear relationship with ORF length (R = 0.86). Furthermore, the parameters of the best fit line also show that, even over the entire range from 300 to 104,000 nucleotides, transcript length closely approximates the ORF length plus a fixed length of approximately 1,263 nucleotides. This is remarkably similar to the relationship between ORF and mRNA lengths in the yeast genome. The difference is the average total UTR length, which is approximately four times longer in humans than in yeast. Furthermore, the average total UTR length estimated by the linear fit agrees well with previous estimates of UTR lengths in human mRNAs of 1,238 nucleotides [14] and 1,070 nucleotides [1]. In order to further characterize the relationship between coding regions and untranslated regions in human mRNAs, we calculated the correlations between ORF and UTR length, 5′-UTR and 3′-UTR length, 5′-UTR and ORF length, and 3′-UTR and ORF length (Table 4). These correlations are all very small and not all positive. This means that the lengths of the three components of an mRNA, the 5′-UTR, ORF, and 3′-UTR, are essentially uncorrelated.

**Table 4:** Correlations between ORF, UTR, and mRNA lengths

Correlation	Refseq	H-Inv
ORF to mRNA	0.86	0.45
ORF to UTR	0.05	−0.24
5′-UTR to 3′-UTR	0.05	−0.03
5′-UTR to ORF	−0.02	−0.24
3′-UTR to ORF	0.05	−0.12

We also performed a similar analysis with the H-Inv database. H-Inv clone records also include coding sequence information, so we were able to calculate the 5′-UTR, ORF, and 3′-UTR lengths for every H-Inv cDNA. Removal of H-Inv clones with 5′- or 3′-UTRs less than 20 nucleotides filtered out nearly half of the clones (42%). We calculated the same correlations as before. As shown in Table 4, the correlations are all quite different between H-Inv and Refseq. Collectively, these results suggest that many of the H-Inv cDNA clones are not full-length. ORF length is negatively correlated with both 5′- and 3′-UTR length. This means that the 5′-UTRs for longer ORFs are generally shorter. Since full-length cDNAs are produced by reverse transcribing mRNA by priming from the poly(A) tail, it follows that putatively full-length cDNAs are more likely to be complete on their 3′-end. Since the primary difficulty in reverse transcribing long mRNAs is the relatively high probability that the reverse transcriptase will fall off the RNA before it finishes transcribing the entire mRNA, it also follows that cDNAs with longer ORFs are less likely to contain complete 5′-UTRs (or even complete ORFs). This is exactly what we observed. The most negative correlation we calculated was between ORF and 5′-UTR length. It should be noted that the presence of significant numbers of incomplete clones in the H-Inv set did not affect the overall correspondence between the lengths from our dataset and the lengths of the H-Inv clones. The genes used in the comparison were represented by an average of three H-Inv clones. As long as any one of those clones was full-length, our length measurement could register a match. We repeated the comparison of our length dataset to the H-Inv clone lengths but with the clones with short UTRs removed. The percentage of length measurements with a match fell 5–6% at all confidence levels, but the probability of a random match fell by a slightly greater amount at all confidence levels, indicating that removal of those clones essentially removed only accidental matches.

### Identification of functionally distinct groups of genes whose UTR lengths deviate significantly from the norm

We investigated whether the length of an mRNA or any of its component sequences has any relationship to the biological role of the protein it encodes. In order to accomplish this, we downloaded the Gene Ontology (GO) annotation (biological process, molecular function, and cellular component) for every clone in our dataset that was associated with a single Entrez gene. We calculated the total UTR length for every length measurement in our dataset that could be associated with a Refseq by subtracting the length of the Refseq's ORF from the length measurement. We then compared the distribution of lengths for every distinct GO classification annotated to at least 10 lengths in our dataset with the distribution of all lengths in the dataset using a Student's t-test (two-tailed, unequal variance). We also performed this analysis using the Refseq lengths themselves. These analyses identified groups of genes with the same GO annotation whose UTR lengths were significantly longer or shorter than average. The results using our length measurements and the Refseq lengths were largely the same. The results were also largely the same across all confidence levels. Tables 5 and 6 show the five most significant GO annotations for UTRs both longer and shorter than average along with their associated ontology and t-statistics (using the 90% confidence lengths).

**Table 5:** Top five GO annotations whose UTRs are significantly shorter than average

GO annotation	t-statistic	Ontology	Refseq t-statistic	Number of lengths
Ribosome	1.83E-30	MF/CC/BP	8.21E-25	152
Mitochondrion	7.84E-18	CC/MF/BP	2.50E-42	351
Glutathione transferase activity	7.01E-08	MF	2.43E-08	21
Proteasome core complex	1.86E-07	CC	2.41E-12	21
Oxidoreductase activity	3.24E-05	MF	3.96E-21	221

Biological process (BP), molecular function (MF), and cellular component (CC).

**Table 6:** Top five GO annotations whose UTRs are significantly longer than average

GO annotation	t-statistic	Ontology	Refseq t-statistic	Number of lengths
Signal transduction, small GTPase	1.03E-08	BP/MF	5.16E-05	137
Intracellular protein transport	2.22E-05	BP	6.17 E-04	122
Ubiquitination	2.60E-05	MF/CC	5.58E-07	136
Membrane	7.60E-05	CC	5.88E-03	428
Regulation of transcription	8.69E-04	BP/MF	3.11E-16	410

Biological process (BP), molecular function (MF), and cellular component (CC).

One remarkable thing about the lists of GO annotations associated with deviant UTR lengths is their similarity to the analogous lists generated for the yeast genome [9]. In both organisms, the UTRs of mRNAs encoding ribosomal, nucleolar, and proteasomal proteins are shorter than average, while the UTRs of mRNAs encoding proteins involved in signal transduction, transcriptional regulation, regulation of metabolism, and cell cycle regulation are longer than average. We previously hypothesized that longer than average UTRs contain additional sequences important for the regulation of the translation, cellular localization, or decay of their mRNA. We believe that the observation that the UTRs of mRNAs encoding proteins of regulatory function are longer than average in man as well as yeast strengthens this hypothesis and points to the importance of post-transcriptional regulation in the fate of mRNAs of eukaryotes in general.

We also searched for GO annotations enriched among genes for which we had identified more than one putative transcript length. We performed a t-test analysis as before. However, no GO annotations showed statistical significance. We then repeated this analysis to search for GO annotations enriched in genes with multiple Refseqs. In this analysis, we identified a small number of GO annotations that had more Refseqs than average. Although these GO annotations were only marginally significant statistically, many of them were involved in signal transduction, specifically protein phosphorylation and dephosphorylation, and transcriptional regulation. A previous analysis of alternative splicing in human [7] also identified genes involved in signal transduction and transcriptional regulation as having higher than average numbers of alternative splice forms.

## Discussion

We applied the Virtual Northern technique to the human genome. Using mRNA purified from human brain as our sample, we obtained provisional length measurements from 21,257 cDNA clones representing a total of 11,536 human genes. Thus, we were able to derive at least one measurement of transcript length for nearly half of the 25,000 genes the human genome is predicted to encode, and from 6,238 of those genes at high (90%) confidence. This is a reasonably high fraction considering that we analyzed mRNA from only a single organ, albeit the organ with the highest transcriptional diversity [12], [13]. Our transcript length dataset has a mean and median of 2,165 nucleotides and 1,996 nucleotides respectively. These numbers agree well with previous estimates for the human genome [1]. At high (≥90%) confidence, only about 1.3% of the clones in our dataset detected two transcript lengths. Current estimates for alternative splicing are that 74% of multi-exon genes have alternate splice forms, and that alternatively spliced genes have an average of 2.7 different splice forms [11], [4]. Our detection rate for alternative transcript variants is expected to fall short of those estimates for two reasons. First, we only examined a single tissue, so our analysis was only able to detect transcript variants expressed in the brain. Second, our length fractionation procedure had a theoretical maximum resolution of about 5–6% of total transcript length. Any transcript variants whose lengths differ by less than that would not be reliably resolved. That range is sufficient to exclude detection of alternative splices resulting from the use of alternate exons of similar length, the inclusion/exclusion of a single short exon, or the use of alternate nearby 5′ or 3′-splice sites.

We compared our transcript length dataset to the lengths of the sequences in the human Refseq database. A total of 23,324 length measurements were compared to 15,104 Refseqs. For length measurements of greater than 90% confidence, 61% matched the length of a transcript recorded in the Refseq database. A rough calculation estimates that the Refseq database currently contains only about 40% of the transcripts encoded by the human genome. With about half of the length measurements in our dataset unrepresented by a Refseq sequence, our data supports that estimate.

Analysis of the transcript length measurements without a matching Refseq showed that the Refseqs from the corresponding gene were longer than our length measurement nearly three quarters of the time. We also observed this bias toward longer Refseqs in our comparison between the Refseq and H-Inv full-length cDNA clone databases. This appears to be due to the inclusion in many Refseqs of all of the sequence from the 5′-most cDNA to the 3′-most cDNA for a given gene. Although this is presumably done to counter the difficulty of identifying full-length cDNAs and to ensure for many mRNAs that the full transcript is represented, it appears that, for many genes, the most extreme boundaries for a transcript are not necessarily the most common. It remains to be determined whether the additional sequence occasionally observed on the ends of transcripts is, for some genes, biologically relevant sequence regulated to occur in only a fraction of transcripts, or if it is merely unregulated, irrelevant transcriptional noise.

We found a striking relationship between human mRNA lengths and the lengths of the ORFs they encode. Comparison of the ORF and mRNA lengths of the sequences in the Refseq database identified a close linear relationship between ORF and mRNA length. Remarkably, this linear relationship has the same form as the relationship we identified between yeast mRNAs and their ORFs, namely that the mRNA length equals the ORF length plus a fixed length of approximately 1,263 nucleotides. Thus in man, as in yeast, the distribution of lengths of untranslated regions in mRNAs does not depend on the length of the ORF.

Several analyses suggested that the H-Inv full-length cDNA clone database contains many clones that are unlikely to be full-length. First, nearly half of the clones in the database have 5′- or 3′-UTRs shorter than 20 nucleotides. In many of these cases the 5′-UTR is completely missing. In contrast to the strong correlation between ORF and mRNA length, and the small correlation between ORF and 5′-UTR length that we identified in Refseq, the H-Inv clones showed a weaker correlation between ORF and clone length and a moderately negative correlation between ORF and 5′-UTR length. This is exactly the relationship we would expect for a set of cDNAs that are not truly full-length, since the primary difficulty in producing full-length cDNAs is obtaining the full 5′-end. Comparison of the H-Inv clones to our mRNA length dataset can now be used as an additional criterion to determine which individual cDNAs are not full-length.

Having a discovered a relationship in yeast between the length of an mRNA's untranslated region and the function of the protein that it encodes, we performed a similar analysis with our human dataset. We were able to identify various functional classes whose UTRs were either longer or shorter than average. Remarkably, there was substantial similarity between yeast and man in this relationship. In both organisms, the UTRs of mRNAs encoding ribosomal, nucleolar, and proteasomal proteins were shorter than average while the UTRs of mRNAs encoding proteins involved in signal transduction, transcriptional regulation, and the regulation of metabolism were longer than average. A recent study of microRNAs extended this observation to *Drosophila*, and suggested that the observed relationship was due to an evolutionary pressure to enrich or deplete microRNA target sites from the mRNAs of certain genes [15]. Ubiquitously expressed genes involved in basic cellular processes appear to have evolved mRNAs with short UTRs, perhaps reflecting a relative paucity of regulation by RNA binding proteins and microRNAs. In contrast, genes involved in more complex regulatory processes such as embryonic development have evolved longer UTRs with many microRNA target sites and perhaps also binding sites for RNA binding proteins, providing an additional layer of regulation. A few functional groups of genes were more likely to have mRNAs of more than one length. Our analysis corroborated a previous analysis of alternative splicing, which identified genes involved in signal transduction and transcriptional regulation as having higher than average numbers of alternative splice forms [7].

## Materials and Methods

### RNA preparation

One whole human brain from a 36 year old male was flash frozen in liquid nitrogen approximately 18 hours postmortem. Brain tissue was provided by Hannes Vogel, Professor of Pathology at Stanford University School of Medicine. Consent was obtained from the donor to use his tissues for research purposes, and the Stanford Medical Board approved their use in this study. The whole brain was crudely pulverized while frozen, and 360 g of tissue was homogenized in a Waring blender containing 38% phenol, 5% glycerol, 10% 1 M sodium acetate pH 4.8 by volume, guanidinium thiocyanate at a final concentration of 0.8 M, ammonium thiocyanate at a final concentration of 0.4 M, and approximately 1 mg/L of the red dye Sudan III. Total RNA was prepared as previously described [16], and poly(A) purified using the Oligotex™ mRNA kit from Qiagen to create the final human brain mRNA sample.

### Length fractionation

A 50 µg aliquot of the poly(A) RNA and a RNA ladder (a mix of the Millennium™ and Century™ markers from Ambion) was heat denatured for 10 minutes at 65°C in formamide, placed on ice, and immediately loaded on a 1.1% low melting point agarose gel in 1X TAE running buffer. The poly(A) RNA was loaded in a wide (5 cm) well to prevent overloading of the gel. The RNA was then separated by electrophoresis at high voltage (∼9 V/cm) for 2–2.5 hours. During electrophoresis, the running buffer was passively recirculated, and the apparatus was cooled to prevent the gel and running buffer from overheating. The ladder lane was excised from the gel, and visualized by ethidium bromide staining. Based on the mobility of the ladder bands, a portion of the poly(A) RNA lane, corresponding to RNAs of approximately 400–7000 nucleotides in length, was cut into 50 slices of 2 mm each. RNA was then recovered from each length fraction by β-agarase digestion. Each agarose slice was melted at 70°C for 10 minutes, and then digested with 2 Units of AgarACE enzyme (Promega) at 42°C for 2 hours. The products from each agarase reaction were recovered using a Microcon-30 column (Amicon) and washed twice with 10 mM Tris pH 7.0 buffer. In total, length fractionation was performed in triplicate using three gels that were run and sliced independently.

### Microarray analysis

One third of the recovered material from each length fraction was reverse transcribed in the presence of amino allyl-dUTP. A mix of dT_20_ and random nonamer was used to prime the reaction in order to maximize the reverse transcription of both polyadenylated and non-polyadenylated RNAs, and to facilitate the labeling of the entire RNA sequence. The resulting cDNA was fluorescently labeled by coupling reactive Cy5 to the amino allyl groups on the incorporated dUTP. Each labeled length fraction was then hybridized to a separate human cDNA microarray. Construction of the human microarrays and hybridization of labeled cDNA to them was accomplished as previously described [17]. Printing of the microarrays was preformed by the Stanford Functional Genomics Facility. To provide an internal hybridization reference, a 1.5 µg aliquot of the human poly(A) RNA was reverse transcribed using the same protocol as the length fractions, fluorescently labeled by coupling the cDNA to Cy3, and included in each hybridization. Microarrays were scanned with an Axon Instruments (Foster City, CA) scanner, and the data collected with GENEPIX PRO 5.1 software (Axon Instruments).

The Cy5/Cy3 fluorescence ratio data was then filtered for quality. Spots with defects apparent from visual inspection, or a coefficient of variation of the signal intensity in either channel greater than 120 were excluded from further analysis. Spots were also filtered on the basis of the background-corrected intensity in the reference channel. The cutoff was determined independently for each microarray by calculating the standard deviations of ratios from duplicated spots. For each microarray, the mean of those standard deviations was calculated with the data from spots with a background-corrected reference channel intensity less than 150 filtered out. If the mean was greater than 0.2, the filter cutoff was increased in increments of 25 until the mean was less than 0.2. For 128 of the 150 microarrays, a cutoff of 150 was used. For the remaining 22 microarrays, the cutoff ranged from 175 to 675. Finally, for each electrophoretic profile based on the 50 measurements for each analyzed DNA sequence, the median background-corrected reference channel intensity (MRI) for all spots in the profile was calculated. Profiles with more than 10 missing data points, or whose MRI was less than 250, were excluded from further analysis. Sets of three electrophoretic profiles based on the same analyzed DNA sequence from each independent length fractionation were excluded from further analysis if any of the individual profiles were excluded.

For normalization between the length fractions, an internal standard was prepared with a pool of *in vitro*-transcribed *Bacillus subtilis* RNAs [18]. PCR products representing five different *B. subtilis* DNAs were printed onto the microarrays and *in vitro* transcribed into RNA. A mix of 400 pg of each *B. subtilis* RNA was doped into each gel slice during the agarase digestion, and into each 1.5 µg reference aliquot before labeling. The internal standard gives us a way to account for differences in the efficiency of RNA recovery between the length fractions. The data from each length fraction was normalized by the value that set the median of the *B. subtilis* ratios to 1.5. Due to slight systematic errors in the normalization of a handful of length fractions, each length fraction was additionally normalized by making a least squares fit of the mean of all ratios from each of the fifty length fractions to a fourth order polynomial, and normalizing the data from each length fraction by the value that set each mean to the polynomial's corresponding value. This normalization “smoothing” had only a minor, fine-tuning effect, modifying each length fraction by only 1.2-fold on average.

In order to facilitate comparison of data between the three separate length fractionations, an internal standard was prepared with a pool of *in vitro*-transcribed *Methanococcus jannaschii* RNAs. PCR products representing approximately 40 regions from the *M. jannaschii* genome ranging from 250–10,000 bps were printed onto the microarrays, and *in vitro* transcribed into RNA. A mix of 400 pg of each *M. jannaschii* RNA was doped into each 50 µg aliquot of the brain mRNA preceding gel electrophoresis, and into each 1.5 µg reference aliquot before labeling. This set of synthetic mRNAs fractionated along with the human brain mRNA gives us a series of profiles with known and consistent characteristics to assist in comparing the data between the three separate length fractionations. Both the *B. subtilis* and *M. jannaschii* RNAs were *in vitro*-transcribed using respectively the T3 and T7 MEGAscript® kits from Ambion.

### Aligning the data from the three gels

Since the RNA from each length fractionation was electrophoresed for a slightly different period of time, the slices from each gel occur at different distances from the well and represent different ranges of RNA length. In order to compare the length profiles for a given gene generated from each length fractionation, it was necessary to scale the data for two of the length fractionations along the gel mobility axis so that they correspond to the data from the third length fractionation. Peaks were manually identified in the profiles of the *M. jannaschii* controls in all three fractionations. Corresponding peaks were compared, and nearly linear relationships between gel mobilities for each of the gels were identified. The least squares fit to a quadratic equation was calculated for each relationship. We applied piecewise cubic spline interpolation to each length profile, and used the above relationships to convert the gel mobilities for the length profiles from gels 2 and 3 into their equivalent mobilities in gel 1.

### Peak finding by *l*_1_ norm baseline deconvolution

In order to identify a small set of underlying peaks within a large set of potentially noisy microarray-derived length measurements, we applied a convolutional model to our data. In an abstract fashion, finding peaks in our length profiles is equivalent to finding the sparsest solution to an underdetermined system of equations: min ||*x*||_0_ subject to *y* = *Ax*, where *y* is an observed measurement, *x* is the underlying signal, *A* is the convolution operator, and ||*x*||_0_ represents the number of non-zeros in the signal. This is a non-convex combinatorial optimization problem, and in general finding the sparsest solution is NP hard. Therefore we solve the problem for the *l*_1_ norm [19], which can be cast as a standard linear program which is convex and tractable, and solved efficiently using general purpose solvers such as simplex and interior point methods [20]. In addition when the solution is sufficiently sparse there is equivalence between the *ell*_1_ and sparsest solutions [21], [22].

In order to account for noise in our data [23], we cast our problem as: min ||*x*||_1_ subject to ||*y*−*Ax*||≤ε. While this model accounts for spurious peaks within noisy data, it is not sufficiently suitable for our purposes. In our case, we would also like to accommodate signals with low frequency content which the deconvolution model represents as multiple peaks. We therefore incorporate a rolling baseline into the model such that the observation *y* is represented as a smooth baseline *u* with peaks on top *v* and noise, and solve the optimization problem: min ||*x*||_1_+*u*||*β*||_1_+½||*r*||_2_^2^ subject to *u*+*v*+*r* = *y*, *Ax* = *v*, and Δ^2^ *u* = *β*. In other words, we solve for the underlying peaks which when convolved with the kernel and added to the smooth baseline result in the observed data. Figure 2 shows an original length profile along with the rolling baseline and the result of deconvolution.

**Figure 6:**
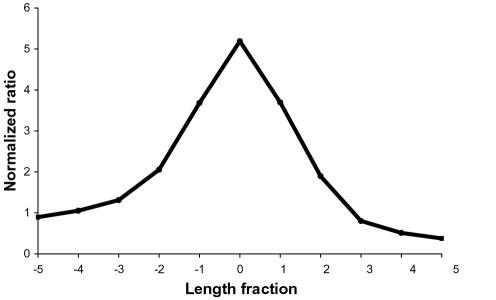
Deconvolution kernel. The stereotypical peak used as the deconvolution kernel to identify potential peaks is shown.

The kernel was derived directly from the data by identifying a large number of likely peaks, lining up those peaks, and taking the median. Peaks were identified in all length profiles by counting each position greater than its two neighboring positions with a normalized ratio greater than four (a high threshold for this dataset) as a peak. The median of the normalized ratio in all of those peak positions was calculated as well as the median of the values of each of the five length fractions to each side of the peak position. This created an 11 element kernel (Figure 6).

### Estimating a confidence value for each peak

In order to ascertain a confidence for each peak, we assigned a probability to each length fraction for every profile. This value describes the likelihood at every position that we have uncovered a true peak at that position. We calculated those probabilities by sampling each value of the deconvolved signal with Poisson noise to create 100 parametric bootstrap replicates. More specifically, given the baseline deconvolution result according to our model, we simulated the experimental data separately at each position by convolution and a Poisson model. Then, we reconstructed each simulation by deconvolution to obtain 100 bootstrap replicates. We counted the fraction of these reconstructions in which there was a peak and computed a probability.

The final dataset of peaks was determined from the bootstrap probability profiles derived from each length profile. First, the results from each of the three gels were combined by averaging the probabilities for each position from each of the three experiments. Every non-zero position in the combined profiles with a probability value greater than its two adjacent positions was called a peak. A precise position for each peak was calculated as the weighted average of each peak position and its two adjacent positions. The probability for each peak was taken to be the sum of the probabilities of the peak position and its two adjacent positions.

### Conversion of gel mobility to transcript length

A “gold standard” set of genes was developed to help evaluate the human Virtual Northern data set. The set consists of 100 abundantly expressed genes that are represented on the microarrays by at least one clone, have at least one identified peak in the dataset, and have at least one curated Refseq [8]. The gold standard genes are derived from the Entrez genes whose associated Unigene clusters have the largest number of clones in them. They are therefore extremely well-sampled by currently existing cDNA and EST databases giving us a far greater than average understanding of the sequence and structure of their mRNAs. Although these genes are all abundantly expressed in at least one tissue, they are not necessarily abundantly expressed in brain. The distribution of microarray signals for the gold standard genes is not significantly different from the dataset as a whole, so they provide a fair representation of the entire dataset, and do not just represent the most well-measured genes in the dataset.

The estimated mobilities of all peaks identified from clones corresponding to gold standard genes were compared to the known lengths of all of the gold standard genes. As expected, the estimated mobilities showed an excellent linear fit to the natural log of the known lengths. Gross outliers (spots deviating from the linear fit by greater than 0.2) were removed, and the least squares fit to a linear equation was calculated (Figure 3). A model of the poly(A) tail was incorporated into the linear equation by adding a fixed length to each known length. A fixed length of 225 nucleotides was calculated along with the slope and y-intercept to provide the best fit between estimated mobilities and transcript length. Using the resulting equation, the estimated mobility of each peak could be converted to an inferred transcript length in nucleotides.

### Calibration of bootstrap confidence value to true peak probability

**Figure 7:**
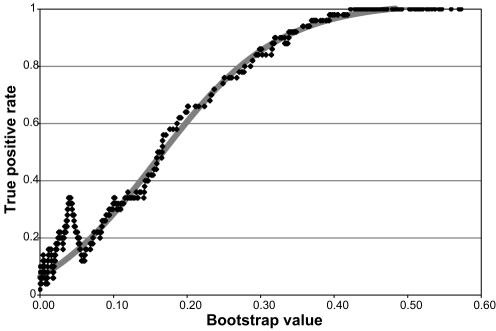
Calibration between bootstrap value and true positive rate. The bootstrap value is plotted against the true positive rate for bootstrap values from 0 to 0.6. The least squares fit to a sigmoidal equation is shown by a solid line with the parameters y = −0.07563+1.09892/(1+e ^(0.15928−x)/0.08257^).

The bootstrap probabilities provide a confidence value for every peak. They are related to, but not equivalent to the probability that a peak is *bona fide*. In order to determine the correspondence between the bootstrap probabilities and the probability that a peak is real, we compared our length dataset to the known lengths from our gold standard gene list. Measured lengths within three fractions of a known length were considered true positives, and all other measured lengths were considered false positives. For each bootstrap value, we calculated the probability that a peak is real as the fraction of peaks at that value that were true positives. The resulting curve (Figure 7) was fit by least squares to the sigmoidal equation y = A+B/(1+e ^(m−x)/l^), and that equation was used to convert bootstrap probabilities into true positive peak probabilities.

### Data Availability

All data is available for download at http://microarray-pubs.stanford.edu/humanVN/.
